# Under Pressure: Shading, High Herbivory, and Low Levels of Fertilization Drive the Vegetative Response of a Highly Invasive Species

**DOI:** 10.3390/plants15030349

**Published:** 2026-01-23

**Authors:** Henrique Venâncio, Guilherme Ramos Demetrio, Estevão Alves-Silva, Tatiana Cornelissen, Pablo Cuevas-Reyes, Jean Carlos Santos

**Affiliations:** 1 Postgraduate Program in Ecology and Conservation, Department of Ecology, Federal University of Sergipe, São Cristóvão 49107-230, Brazil; henrivens@gmail.com; 2 Laboratory of Plant Ecology, U. E. Penedo, Campus Arapiraca, Federal University of Alagoas, Penedo 57200-000, Brazil; guilherme.ferreira@penedo.ufal.br; 3 Federal Institute of Education, Science and Technology, Urutaí 75790-000, Brazil; estevao.alves@ifgoiano.edu.br; 4 Department of Genetics, Ecology and Evolution, Federal University of Minas Gerais, Belo Horizonte 31270-901, Brazil; taticornelissen@gmail.com; 5 Laboratory of Ecology of Biotic Interactions, Michoacana University of San Nicolás de Hidalgo, Morelia 58004, Mexico; pcragalla@gmail.com; 6 Department of Ecology, Federal University of Sergipe, São Cristóvão 49107-230, Brazil

**Keywords:** phenotypic variation, competition, fertilization, simulated herbivory, fluctuating asymmetry

## Abstract

Invasive plant species persist under environmental conditions due to phenotypic plasticity, which allows them to cope with conditions such as herbivory, competition, and resource availability. However, plant responses to individual and combined stressors are variable. In addition, fluctuating asymmetry (FA) has been proposed as an indicator of plant stress, although its reliability remains debated, and few studies have evaluated its responses under interacting stressors. We evaluated, in two greenhouse experiments, the isolated and combined effects of herbivory and shading; and belowground intraspecific competition and fertilization on performance, trait plasticity, and leaf FA in seedlings of the invasive plant *Tithonia diversifolia*. Shading reduced shoot biomass, but promoted plastic adjustments in architectural, photosynthetic, and leaf structural traits that enhance light capture, and also increased FA. Herbivory interaction with shade induced high leaf mass per area of plants. In contrast, high herbivory and intraspecific competition consistently reduced plant performance across multiple traits. Fertilization enhanced overall performance and mitigated the negative effects of herbivory and competition. Overall, our results emphasize the need to consider interacting environmental factors when assessing invasive plant performance and plasticity. Furthermore, FA showed inconsistent responses across treatments, suggesting its limited reliability as a biomarker of isolated and combined environmental stress.

## 1. Introduction

Invasive plant species are widespread in natural and human-modified environments, and their diffusion has expanded drastically in recent years due to increasing human activities and climate change [1]. Despite their environmental tolerance, invasive plants exhibit high phenotypic plasticity, enabling rapid and flexible responses to unfavorable conditions [2,3]. Such plasticity allows invasive plants to compete with native species and to persist across diverse environmental conditions, facilitating their success in natural and anthropized environments [3,4].

Invasive plants may experience herbivory, at low or high levels, either naturally or through biological control in invaded habitats [5]. Herbivory can reduce invader performance, and, at high levels, compromise population viability [6,7]. In response, plants may exhibit resistance and tolerance strategies. For instance, plants may maintain or enhance the performance of key traits through toleration and compensatory responses to leaf loss [8]. Alternatively, herbivore attack may be reduced by the induction of chemical and structural anti-herbivory defenses [9]. Despite extensive research on herbivory responses in invasive plants, the effects on performance and defensive strategies vary widely among species and across environmental conditions [10,11,12].

Plants may invade environments where local vegetation limits light availability to understory individuals. This condition is detrimental to shade-intolerant species and reduces both establishment and individual performance [13]. However, there is growing evidence that some shade-intolerant invasive species successfully establish under closed-canopy or in gap environments [14,15]. To cope with aboveground competition, plants optimize their architecture and physiological traits to maximize light capture, such as stem elongation and photosynthetic optimization [16]. However, when herbivory occurs simultaneously, a trade-off between shade-induced and anti-herbivory responses may occur [17], as one may limit or counteract the morphological and physiological adjustments to the other [18,19,20]. For instance, plants may reduce leaf structural reinforcement to enhance longevity and light absorption under shading [21], although this response may vary across growth forms and environmental conditions [22]. This light capture response is antagonistic to the leaf toughening anti-herbivory strategy used by some invasive plants [23]. Moreover, some invasive plants can tolerate herbivory under shade conditions without marked declines in plastic responses to low light conditions [24]. The effects of herbivory and shade interactive plastic responses have been extensively studied in native plants, but remain poorly explored in invasive species.

Once established, invasive plant populations might be locally abundant, leading to intense belowground intraspecific competition [3]. Intraspecific competition for soil resources constrains plant performance [25], although some dense invasive populations show no significant negative effects [26,27,28]. Consequently, resource limitation may exacerbate the negative effects of other stressors, such as herbivory [29]. Particularly, plastic responses to herbivory are energetically costly [30] and are limited under resource limitation [31,32]. Conversely, resource supplementation can mitigate these negative effects. Soil fertilization can increase nutrient uptake and allocation to vegetative and reproductive traits in invasive species [33,34]. Moreover, fertilization can mitigate the effects of competition and herbivory by enabling tolerance or compensation for resource limitations [35,36,37]. Although studies have assessed the effects of these three factors individually or in combination, most have focused on invasive performance or invasive–native interactions, leaving the combined impacts of these on plasticity poorly understood.

Plastic responses to stressors can disrupt cellular development (e.g., cell division and growth), generating subtle instabilities in the symmetrical structures of organisms [38]. Fluctuating asymmetry (FA) is the small and random deviations from perfect bilateral symmetry, and is widely used as a bioindicator of environmental stress [39]. Due to its rapid assessment, low cost, and straightforward interpretation, FA has been consistently used as a measure of environmental and biotic stress in animals, algae, and especially plants [40]. In plants, FA has been subjected to frequent methodological refinement [41], reinforcing the usefulness of this technique to evaluate stress response [42,43].

Most studies, however, relate FA to a single stressful condition, and rarely investigate how multiple factors affect plant development [44]. For instance, [45] found that leaf FA increased under combined exposure to heavy metals and ionizing radiation; in contrast, [46] showed that only one of two stressors (drought vs. temperature) affected leaf FA. When biotic and abiotic factors are combined, biotic stressors often appear to increase FA (pollution vs. herbivory—[47]; moisture vs. herbivory—[48]). Nonetheless, in factorial designs interaction effects and confounding factors may arise between biotic and abiotic conditions [49,50].

Herbivory can largely affect plant performance such as growth, biomass allocation, and reproduction [51]. Thus, a positive (cor)relation between FA and herbivory is often expected, but this is not always the case [52,53]. This occurs because FA can covary with other factors, and some plants may tolerate herbivory, resulting in no detectable increases in FA levels [54]. For instance, shading is one factor frequently evaluated in combination with herbivory in FA studies. When examined individually, understory plants show higher FA under sunny conditions, and vice versa [50,55]. However, when herbivory and shading were experimentally combined, FA was higher in the shade, but herbivory increased the FA of sun-exposed plants [49]. These contrasting patterns highlight the context dependency of FA.

In this study, we tested how multiple environmental pressures (such as herbivory and competition) affect the performance and plastic response of invasive plants. We used the shade-intolerant invasive *Tithonia diversifolia* (Hemsl.) Gray (Asteraceae) as a study model because its strong invasive ability, high phenotypic plasticity, and rapid FA responses make it ideal for testing how different environmental pressures affect plant development [56,57]. We combined simultaneous environmental conditions in two experiments: one integrating herbivory and shading, and another combining herbivory, belowground competition, and fertilization. Specifically, we addressed three main questions: (i) How do herbivory, shading, and belowground competition, alone and in combination, affect vegetative traits, including the architecture, biomass allocation, and leaf traits of *T. diversifolia*? (ii) Does fertilization mitigate the negative effects of herbivory and belowground competition on this plant species? (iii) Can fluctuating asymmetry (FA) serve as a sensitive biomarker of stress under single and combined environmental factors? Overall, we hypothesized that *T. diversifolia* exhibits context-dependent shifts in performance and plastic responses under both isolated and combined stressors interacting with resource availability. We predicted that (i) herbivory would reduce plant performance by changing the plant architecture, biomass allocation, and leaf functional traits, while increasing leaf structural toughness. In contrast, we expected shading to induce shade-avoidance responses in increased plant architecture, greater investment in aboveground biomass, enhanced light capture leaf traits, and reduced leaf toughness. When combined, herbivory-induced responses were predicted to constrain shade-induced trait adjustments. In the second experiment, we expected that belowground competition would reduce overall plant performance, with stronger negative effects when combined with herbivory. (ii) We further predicted that fertilization would mitigate the negative effects of herbivory and belowground competition across traits by increasing plant tolerance and reducing leaf toughness. (iii) Finally, we hypothesized that fluctuating asymmetry would be an effective biomarker of environmental stress, increasing progressively as the number of interacting stressors increases. By examining multiple stressors and fertilization, our study offers new insights into invasive plant performance, highlighting not only changes in growth and functional traits but also developmental instability (FA) and stress signaling responses.

## 2. Results

### 2.1. Effects of Herbivory and Shading on T. diversifolia

**Figure 1:**
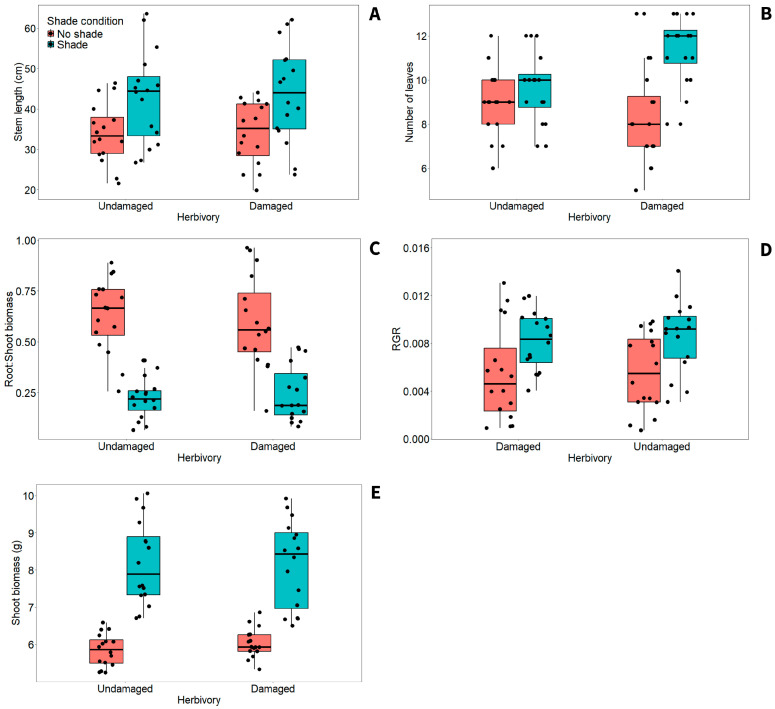
Boxplots showing effects of herbivory and shade condition on (**A**) stem length, (**B**) number of leaves, (**C**) root/shoot biomass, (**D**) relative growth rate (RGR), and (**E**) shoot biomass of *Tithonia diversifolia*. Boxes show the 25th and 75th percentiles, horizontal lines the median, whiskers represent the 10th and 90th percentiles.

Herbivory alone had no effect on architecture and biomass traits (i.e., stem length, number of leaves, shoot biomass, root/shoot biomass, and relative growth rate), but shade condition strongly influenced them (*p* < 0.001; Table 1). On average, shade strongly increased the stem length, number of leaves, and growth rate, while it reduced shoot biomass and root/shoot biomass ratio compared to the sun group (Figure 1). Interactions between herbivory and shade condition also revealed that damaged and shaded plants had considerably more leaves than no-shaded plants at both herbivory level (Tukey: *p* < 0.001; Figure 1A).

**Figure 2:**
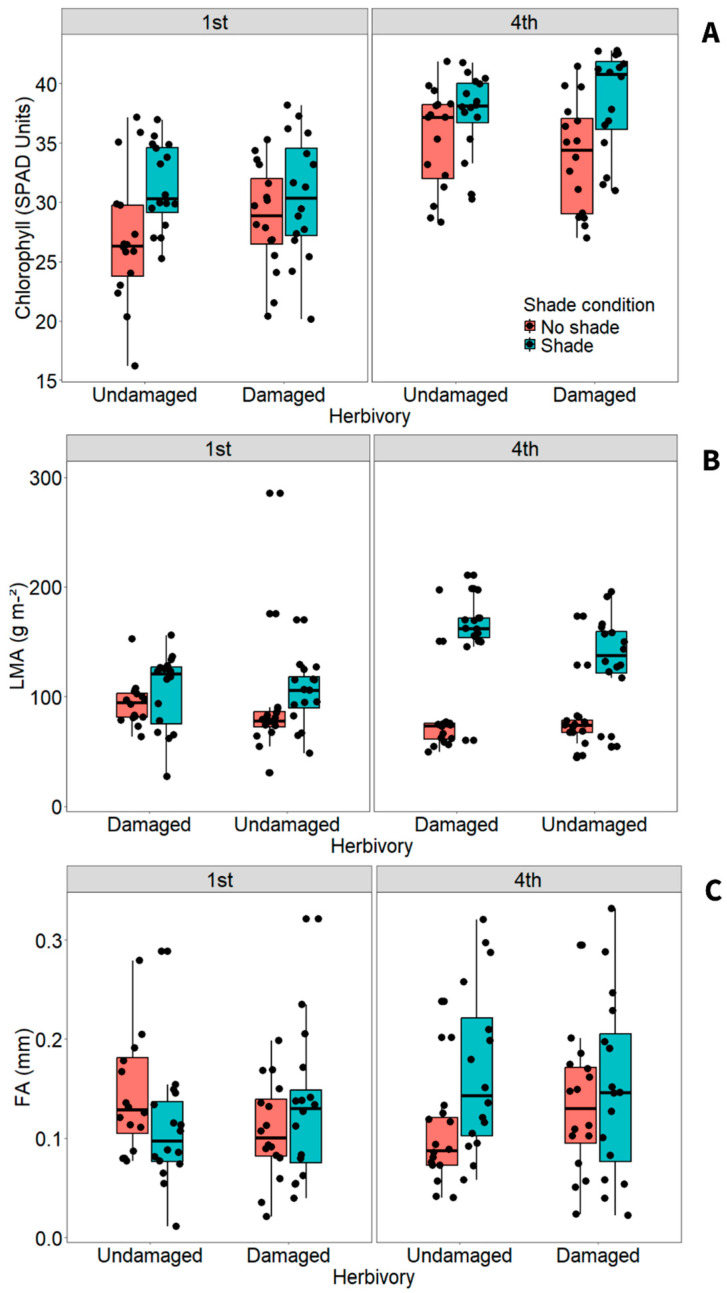
Effects of herbivory, shade condition, and study weeks on (**A**) chlorophyll, (**B**) leaf mass per area (LMA), and (**C**) fluctuating asymmetry (FA) of *Tithonia diversifolia*. Boxplots show data variation: boxes indicate the 25th and 75th percentiles, horizontal lines indicate medians, whiskers indicate 10th and 90th percentiles.

Shade was the dominant driver of vegetative and leaf-level responses, while herbivory alone had comparatively minor effects. Overall, herbivory and shading had no effect on leaf chlorophyll (Table 1), but chlorophyll increased from first to fourth week leaves (*p* < 0.001; Figure 2A). Shade condition noticeably increased chlorophyll from first week to fourth week leaves (*p* = 0.001), especially compared with no shade plants (Tukey: *p* < 0.01). A significant three-way interaction showed that undamaged, shaded plants in fourth week leaves had slightly more chlorophyll than damaged, no shade plants in first week leaves (Tukey: *p* < 0.01). This interaction indicates that the effects of herbivory and shading on chlorophyll are strongly mediated by leaf developmental stage, with newly formed leaves under shade showing greater physiological adjustment over time. Herbivory and shading also affected LMA (Table 2). Undamaged plants had slightly less LMA than damaged ones (*p* = 0.02; Figure 2B), while this trait was highly lower in shaded plants than those in no shade condition (*p* < 0.001; Figure 2B). Shade condition affected LMA across first week and fourth week leaves (*p* < 0.001), indicating that no shade plants had higher LMA in the fourth week, and than shade plants across both weeks (Tukey: *p* < 0.001). In contrast, LMA in shade plants was greater in the first than fourth week (Tukey: *p* < 0.002).

As observed for other leaf traits, shade condition altered the FA of plants (*p* = 0.049; Table 2). On average, shaded individuals were slightly more asymmetrical than shaded plants (Figure 2C). Fluctuating asymmetry also varied across leaves of the two weeks in response to shade condition (*p* = 0.014), where the fourth week leaves of shade plants were moderately more asymmetrical than first week leaves, and more than the fourth week leaves of no shade plants (Tukey: *p* < 0.04). This pattern suggests that leaves initiating and completing development under shaded conditions experience greater developmental instability than those exposed to shade later in ontogeny.

### 2.2. Effects of Herbivory, Edaphic Competition, and Fertilization on T. diversifolia

**Figure 3:**
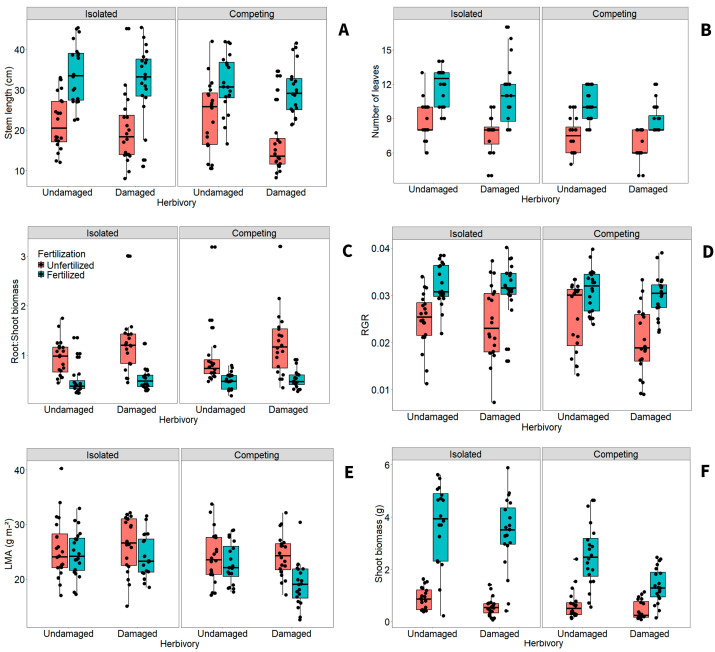
Effects of herbivory, belowground competition, and fertilization on (**A**) stem length, (**B**) number of leaves, (**C**) root/shoot biomass, (**D**) relative growth rate (RGR), (**E**) leaf mass per area (LMA), and (**F**) shoot biomass of *Tithonia diversifolia*. Data variation is shown with boxplots: boxes indicate the 25th and 75th percentiles, horizontal lines indicate medians, whiskers show the 10th and 90th percentiles, and black dots represent outliers.

Herbivory significantly influenced architecture and biomass traits (*p* < 0.037), except LMA (*p* = 0.28; Table 3). Damaged plants had slightly lower stem length, number of leaves, RGR, and shoot biomass than undamaged individuals (Figure 3A,B,D,F). On the other hand, undamaged plants showed higher root/shoot ratios compared to damaged plants (Figure 3E). Similarly to herbivory negative effects, competition level altered the number of leaves and shoot biomass (*p* < 0.001; Table 3), where isolated plants had more leaves and biomass than those growing under competition (Figure 3B). Additionally, competition also influenced LMA (*p* < 0.001; Table 3), showing that this trait was slightly greater in isolated individuals than those under competition (Figure 3E). Fertilization altered all measured traits (*p* < 0.001; Table 3). All traits increased substantially in fertilized plants, whereas the root/shoot ratio and LMA were more than 10% lower in this group (Figure 3), in contrast to the patterns observed under herbivory and competition treatments.

Significant interactions between fertilization and other treatments also influenced plant traits (*p* < 0.01; Table 3). Fertilization slightly reduced the root/shoot biomass in damaged plants and those under belowground competition (Tukey: *p* < 0.05; Figure 3C). Moreover, herbivory and fertilization interactions also showed that fertilized plants presented less LMA in damaged plants (Tukey: *p* < 0.05; Figure 3C). Shoot biomass responded to herbivory and fertilization, and to competition and fertilization interactions (*p* < 0.001). In the first interaction, damaged and fertilized seedlings had considerably less biomass compared to non-damaged and fertilized ones, but the first group strongly had more biomass than unfertilized plants regardless of the herbivory level (Tukey: *p* < 0.001; Figure 3F). In competition and fertilization interactions, fertilized seedlings presented more shoot biomass than unfertilized ones regardless of the competition condition, and fertilized isolated plants also presented more biomass than fertilized and competing ones (Tukey: *p* < 0.001; Figure 3F). Overall, these results suggest that fertilization mitigates the negative effects of herbivory and competition on some vegetative traits.

**Figure 4:**
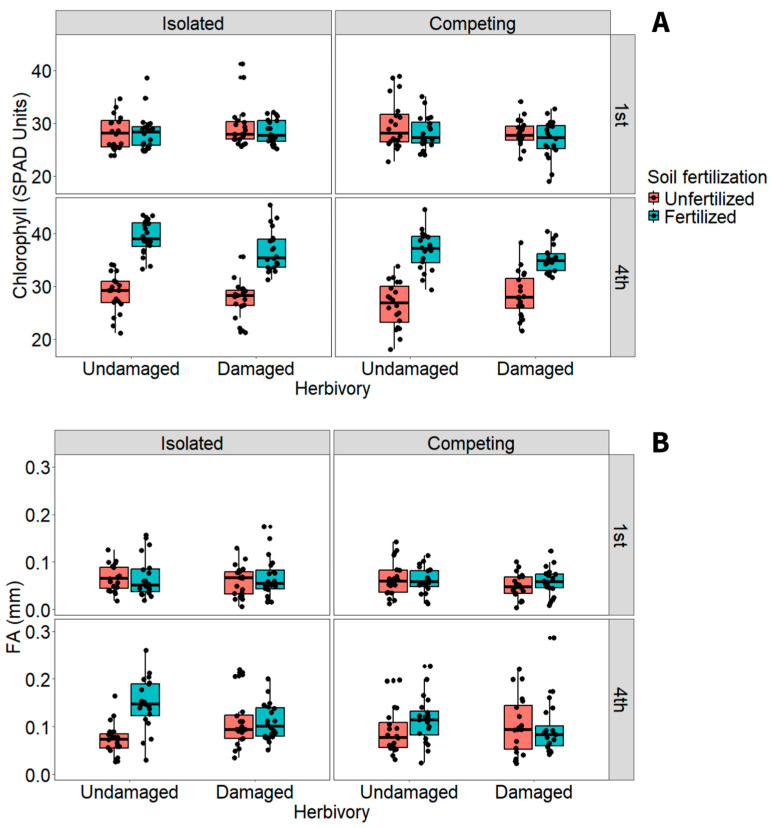
Response of (**A**) leaf chlorophyll and (**B**) fluctuating asymmetry (FA) of *Tithonia diversifolia* to herbivory, soil fertilization, belowground competition, and leaves of the 1st and 4th week of the experiment.

Herbivory did not affect leaf chlorophyll content, but competition, fertilization, and leaves from the first and fourth weeks did (*p* < 0.05; Table 4). Specifically, competing plants had slightly less chlorophyll than isolated ones (Figure 4A). In contrast, fertilization considerably increased chlorophyll compared to unfertilized plants (Figure 4A). The interaction of herbivory, competition, and sampling week reduced chlorophyll by at least 10% in competing, damaged plants in the leaves of the fourth week compared to undamaged, isolated plants in the leaves of the first week (Tukey: *p* < 0.02; Figure 4A). Interestingly, these results suggest that herbivory amplifies the negative effects of competition on chlorophyll content in newly emerged post-treatment leaves.

Leaf FA was significantly influenced by fertilization and the leaves of each week (*p* < 0.004; Table 4). Fertilized plants showed substantially higher FA than unfertilized ones (Figure 4B), and FA was also higher in leaves of the fourth week than those of the first week (Figure 4B). This result is particularly interesting because fertilization, which increased overall plant trait performance, also increased leaf asymmetry, suggesting a potential trade-off between rapid growth and developmental stability. The interaction between herbivory and fertilization showed that undamaged, fertilized plants had slightly higher FA than damaged, fertilized ones (Tukey: *p* < 0.02; Figure 4B). Fertilization also increased asymmetry by over 10% from the first to the fourth week, with a slightly additional increase in undamaged, fertilized plants during the same period (Tukey: *p* < 0.04; Figure 4B). Therefore, the interaction between fertilization and herbivory revealed an unexpected response pattern, with asymmetry increasing in fertilized, undamaged plants.

## 3. Discussion

Our greenhouse experiments showed that isolated and combined simulated environmental conditions induce different effects on the performance and plastic response of *T. diversifolia* seedlings. In the first experiment, which combined low herbivory and shading, we observed plastic changes mainly in architectural, physiological, and stress-bioindicator (FA) traits, consistent with adjustments that enhance light capture. In the second experiment, which combined high herbivory, intraspecific belowground competition, and fertilization, plant responses ranged from reduced performance to pronounced phenotypic shifts and tolerance in almost all traits. Overall, these findings suggest that the phenotypic variation in *T. diversifolia* enables it to cope with multiple biotic and abiotic pressures, supporting its persistence across diverse environments.

### 3.1. Plant Performance and Plasticity Under Herbivory and Shade

Invasive plants can withstand substantial levels of herbivory without incurring fitness costs [57]. We confirmed this characteristic in our study, where ~2% mean leaf area removal did not affect the architecture and biomass traits of *T. diversifolia* seedlings. Moreover, chlorophyll content was not affected by herbivory, suggesting that tolerance does not constrain this physiological trait in this species. Low herbivory, such as 3%, may negatively affect the performance of plants [8], but invasive species may tolerate considerable damage due to some mechanisms, such as substantial resource reserves and high resource use efficiency [58,59]. This characteristic may contribute to enhancing the population performance of invasive plants in enemy-release habitats, where low herbivore damage is predominant, potentially allowing available energy to be allocated to growth and reproduction [60].

The resource-conservative strategy may also explain the non-significant declines in performance despite the activation of anti-herbivory defenses. Despite low herbivory damage, newly formed leaves after simulated herbivory showed high LMA. Tough leaves reduce herbivore palatability and performance, which may reduce leaf removal of plants by herbivores in invaded areas [61]. To our knowledge, there are no reports of mechanical anti-herbivory defense for *T. diversifolia*, but we speculate that this species may increase leaf toughness in concert with chemical defenses to reduce herbivory by generalist insects [57].

Shading reduced biomass accumulation, indicating that this is a negative condition for the performance of *T. diversifolia* seedlings. However, we also observed clear plastic responses to shading, expressed through vegetative and physiological traits that maximize light capture; specifically, increased stem length, leaf number, RGR, and chlorophyll content, combined with a lower root/shoot ratio and LMA. The plasticity of these traits indicates greater investment in aboveground architectural and leaf morphological and physiological traits that facilitate access to more illuminated vertical strata which enhance photosynthetic efficiency under light limitation [62,63]. Together, these responses characterize the shade-avoidance syndrome in this study species, expressed as morphological and physiological adjustments that enhance light acquisition under shade conditions [16], as reported for other shade-intolerant invasive species [64,65]. Although seedlings displayed plastic responses to low light, their reduced performance suggests potential limitations under deep shade. Some invasive shade-intolerant populations may adapt and persist in prolonged and intense shading habitats [14]. Despite previous records of *T. diversifolia* at forest edges [66], the short duration of our experiment (four weeks) and its focus on seedlings limit robust inferences about this species’ potential to invade forest ecosystems [67]. Thus, future studies should investigate the extent to which plasticity enables this species to persist under severe shading.

Seedlings in herbivory and shading treatments showed higher number of leaves compared to those in no shade regardless of herbivory level. This result suggests an allocation on leaf production to compensate for defoliation through the production of additional photosynthetic tissue under shade, in order to maintain maximal light capture [68]. In relation to chlorophyll, undamaged and shaded seedlings presented higher content on newly formed leaves compared to damaged and no-shaded individuals. This may indicate a tolerance strategy, where seedlings maintain chlorophyll concentration in new leaves under shaded conditions despite low herbivory [69]. Overall, our findings suggest that plastic responses to herbivory and shading vary among traits in invasive plants, ranging from tolerance to compensation, thereby contributing to the persistence of this group under variable environmental conditions.

The relationship between herbivory and FA has been extensively investigated in the literature, although many findings remain inconclusive [70,71,72]. In our study, low herbivory levels also did not induce increases in leaf FA. Reference [56] showed that small amounts of herbivore damage were sufficient to increase FA during leaf expansion in *T. diversifolia*. However, the asymmetry levels of seedlings were similar to control plants, both in treated leaves and in subsequently developed ones. This outcome may be explained by the considerably lower damage level (~2%) compared with that reported by [57] (mean of ~4%). This suggests that a minimum damage threshold may be required to trigger sufficient stress to increase FA in developing leaves and in those formed afterward [54]. Such an explanation is plausible given that we observed no effects of herbivory on plant plasticity or performance in our study suggesting that seedlings can buffer herbivory at low levels.

Shaded plants were more asymmetrical than those in the sun, showing that low light disrupts leaf development in *T. diversifolia* seedlings. Together with the reduced biomass and plastic response observed under shading, our results suggest that seedlings of shade-intolerant plants experience high stress, resulting in low performance associated with elevated FA levels [49,54]. Moreover, the higher FA levels observed in newly formed leaves of shaded seedlings, compared with initial leaves under both shading conditions, indicate that leaves initiating development under shade experience stronger stress than those exposed to shading later during mid ontogeny. Similarly, within individuals, leaves developing under shade showed higher FA than those formed under full sun. As suggested by other studies, this condition may indicate that FA may reflect the plastic developmental response of plants to shading rather than an intrinsic indicator of stress [40,73]. Given the uncertain nature of FA, we cannot precisely associate asymmetry with performance or plasticity in our study. Thus, future research needs to clarify how shading indeed influences developmental instability.

### 3.2. Plant Performance and Plasticity Under Herbivory, Competition, and Fertilization

In this experiment, seedlings were subjected to an intense simulated herbivory treatment applied over two weeks, with 100% of the leaf area removed during the first week and 50% of the newly produced leaf area removed during the second week. In contrast to the low levels results of experiment 1, high herbivory negatively affected all measured architectural and biomass traits of seedlings. Although our damage was simulated, this high-herbivory treatment resembles biocontrol scenarios applied to some invasive plants, in which herbivores can rapidly defoliate seedlings and adults almost completely, thus decreasing population persistence in invaded sites [74]. Specifically for *T. diversifolia*, some natural enemies have shown promising results in population control, although many are exotic and either attack non-target plants or fail to establish viable populations [75].

Herbivory did not affect chlorophyll content or LMA, despite the clear decline in seedling vegetative performance. The mismatch between growth traits and chlorophyll suggests that this species prioritizes toleration of herbivory on some physiological attributes that enhance survival [76], even when vegetative performance is compromised. In this sense, we speculate that energy produced by the photosynthetic apparatus may be redirected toward other fitness-related traits, for example [77]. The relationship between vegetative traits and chlorophyll should be further investigated, as this association may be species-dependent [78].

While we observed higher LMA in response to low herbivory in experiment 1, this pattern did not occur under high herbivory. This result suggests that, under intense damage, *T. diversifolia* does not invest in mechanical defenses such as leaf stiffening, and may instead rely on other anti-herbivory strategies, including the induction of chemical compounds [79,80]. Although induced chemical defenses are energetically costly, they can be more effective in protecting early-stage plants [81,82]. Alternatively, other physical defenses not assessed in this study (e.g., trichomes) may also be used by *T. diversifolia*, as they can more directly reduce attacks by chewing herbivores [83].

Invasive plants often exclude neighbors by releasing allelopathic compounds or by developing competitive root systems [3,5,84]. Yet, in some cases, intraspecific competition among invasive individuals is high [85]. In our study, intraspecific competition reduced shoot biomass, leaf number, and chlorophyll content, but not other architecture traits in seedlings. This pattern may indicate that this species prioritizes maintaining its energetic reserves on architecture and biomass at the expense of photosynthetic mechanisms to remain aboveground competition [13,16]. Herbivory interacting with competition further reduced the chlorophyll of newly formed leaves, indicating that this trade-off may also occur under combined stress. Dense populations of *T. diversifolia* prioritize asexual reproduction when key resources, such as water, become limited [86]. Therefore, it is also possible that this species prioritizes the available energy for vegetative growth under competition stress during the seedling stage.

High soil nutrient availability enhances the performance of invasive species [33], and our results are consistent with this pattern. Seedlings of *T. diversifolia* showed strong responses to fertilization, with higher performance across all aboveground traits assessed. This response was expected, as many fast-growing invasive plants are able to capture large amounts of nutrients and rapidly convert them into vegetative growth and physiological processes that enhance the invasion of ecosystems [33,60,87]. Moreover, fertilized seedlings developed more leaves with higher chlorophyll content and lower LMA. This may indicate that this species invests in rapid growth and enhanced photosynthetic capacity to capture light and occupy aboveground space to the detriment of reduced structural toughness [35,88].

Resource use efficiency may also explain why fertilization mitigated the effects of herbivory and competition on aboveground biomass. In invasive species, such resource availability is crucial for tolerating herbivory and suppressing neighboring plants, as it enables individuals to reach the reproductive stage more rapidly and intensely, further increasing their invasive potential [5,89,90]. Moreover, nutrient enrichment may also favor investment in growth rather than in anti-herbivory defenses [91]. This pattern may occur in *T. diversifolia* seedlings, as fertilized plants that were damaged showed reduced LMA. This reduction suggests that the available nutrients are being directed toward increasing growth or replacing lost leaf tissue, including in newly formed leaves, rather than enhancing leaf toughness.

Despite low seedling performance, our results provide further evidence that neither herbivory nor intraspecific competition increases FA, whether applied alone or in combination. Recent experimental studies also found no association between low performance to these or other stressors and asymmetry [92,93], and suggested alternative and more reliable indicators of plant stress [72]. Although FA is widely used as a stress indicator, its association with some environmental stress remains inconsistent, in part due to earlier studies that failed to investigate developmental instability assumptions and may have produced false positives [40]. Therefore, further experimental studies are needed to investigate the validity of FA, particularly in response to stressors such as competition, for which evidence remains scarce, and herbivory, for which results are contradictory in the literature.

Fertilization, in contrast, markedly increased the FA of seedlings. Rapid growth may disrupt the synchronized development of bilateral structures, increasing the window for developmental errors to accumulate and thereby elevating FA [48]. Considering that many plants can efficiently uptake and assimilate soil macronutrients [59], this disruption may explain the high FA levels observed in *T. diversifolia* and in other plant species growing in nutrient-enriched soils [94,95]. However, fertilization enhanced seedling performance, thereby obscuring the expected association between FA and environmental stress. Such an association becomes even more intriguing when we examine the FA response of seedlings under the combined effects of herbivory and fertilization. Seedlings without herbivory but fertilized had higher FA in first and fourth week leaves than those simultaneously exposed to herbivory and fertilization. We hypothesize that our findings reflect developmental leaf plasticity induced by fertilization, rather than a stress-related FA response [40]. Further studies should disentangle whether favorable growth conditions truly increase developmental instability or merely reflect plastic trait responses.

## 4. Materials and Methods

### 4.1. Study Species

*Tithonia diversifolia* is a fast-growing pioneer shrub native to North and Central America that can reach up to 5 m in height [96]. From the seedling stages onward, its leaves gradually develop from one- to five-lobed along plant ontogeny, and are short-lived, reaching ~15 cm and senescing within 45 days after emergence [56,97]. This species grows and develops rapidly, and reproduces both vegetatively and sexually, producing more than 100 seeds per capitulum [86,97]. Together, rapid plant development and short leaf lifespan make this species ideal for quickly investigating how environmental stressors affect plant performance and plasticity, allowing tracking of leaf development over time as new leaves emerge [56]. The rapid development of this plant species contributes to its worldwide distribution and aggressive invasion across Africa, Australia, Asia, and South America [57,98], where it forms dense populations in disturbed sites (e.g., roadsides and agricultural fields), but it can also colonize natural areas, including forest edges [57,99,100]. Although *T. diversifolia* is difficult to manage, it is attacked by several herbivores, some of which have been considered potential biocontrol agents in agricultural areas [75].

### 4.2. Seed Collection and Plant Rearing

We collected approximately 300 seeds of *T. diversifolia* from 50 healthy adult individuals in Uberlândia, Minas Gerais, Brazil (18°52′14″ S, 48°12′50″ W), to use in the experiments. We kept seeds in closed pots for 8 weeks to mature [101] and transplanted them into seedling trays containing Bioflora^®^ substrate (mixture of eucalypt bark, peat, vermiculite, and coconut fiber). After 2 weeks, we randomly transplanted 250 seedlings into polyethylene pots (1.7 L for experiment 1, 500 mL for experiment 2) and acclimated them for 1 week. Six-week-old seedlings were used in experiment 1 and three-week-old individuals in experiment 2. Plants were spaced approximately 10 cm apart in the pots, and the experiments were conducted at different times.

The experiments were conducted in a greenhouse (16 × 6 m) at the Federal University of Uberlândia, Brazil (18°53′10″ S, 48°15′37″ W). The mean temperature of the site was ~31 °C at midday, with a photoperiod of 12 h/12 h. The irrigation was automated to work for 10 min, four times daily, at 6 h intervals. No external conditions (e.g., shading and insects) affected the greenhouse interior.

### 4.3. Experimental Design

#### 4.3.1. Experiment 1—Herbivory and Shading

In the first experiment, we investigated the effects of defoliation and shading on *T. diversifolia* seedlings, simulating an invasion in shaded environments with reduced understory light, such as forest interior. Additionally, we applied low-level herbivory damages on plants, consistent with the ~5% leaf blade removal reported for exotic Asteraceae in some invaded environments [102].

We randomly selected 64 six-week-old seedlings and assigned them to a 2 × 2 factorial design with herbivory (undamaged vs. damaged) and light condition (shading vs. no-shading) as factors. Herbivory treatments were systematically alternated within each light environment to minimize positional effects, totaling 16 individuals per group. Prior to treatment application, we assessed the uniformity of some traits of plants [56]: stem length, number of leaves, leaf length, chlorophyll content, and leaf FA. Generalized Linear Models (GLMs) indicated that these traits were similar among the treatments (*p* > 0.1) (see Table S1 for mean values and standard errors of all measured traits). We measured leaf length, chlorophyll, and FA from three apical leaves per plant. Detailed trait and FA measurement methods are described in the “Trait sampling” section.

After confirming uniformity among the groups, we performed herbivory and light competition treatments. First, we marked the previously evaluated apical leaves (~40% of total leaves, ~2 weeks old) in all plants using a permanent marker. Next, we simulated herbivory on treatment plants by removing ~57 mm^2^ of leaf area per leaf using a 6 mm diameter hole-punch (two holes per leaf; ~2% area removed), while control plants remained undamaged (Figure S1a,b). To simulate light competition, we placed treatment plants under a black shade cloth that intercepted ~80% of solar radiation, whereas control plants were exposed to full sunlight for 12 h per day (Figure S1c,d). Although there are no reports of *Tithonia diversifolia* occurring under such high shading, in the study region some individuals are found in the forest interior where light intensity is very low (personal observation).

We conducted this experiment over four weeks (28 days), from March to April of 2016, before complete senescence of the marked leaves. At the end of the study, we sampled three previously marked leaves and three newly expanded apical leaves per plant (n = 192 leaves per week), representing at least half of the total leaf number per individual. For the sake of clarity, we hereafter refer to leaves marked during the first week of the study as “1st week leaves” and those marked at the end of the study as “4th week leaves”. All sampled leaves were three-lobed.

#### 4.3.2. Experiment 2—Herbivory, Belowground Competition, and Fertilization

In this experiment, we simulated colonization by *T. diversifolia* in fertilized soil and under intense herbivory with biocontrol agents. We focused on three common conditions in post-harvest arable and disturbed natural systems: belowground intraspecific competition due to dense plant colonization, herbivory by chewing insects, and soil nitrogen enrichment [103,104]. Because biocontrol agents can heavily defoliate this plant in the field [75], we simulated intense herbivory in this experiment.

We assigned 160 two-week-old seedlings to a 2 × 2 × 2 experimental setup with soil competition (isolated vs. competing), fertilization (unfertilized vs. fertilized), and herbivory (undamaged vs. damaged) as factors, resulting in 20 plants per treatment combination. We systematically alternated the treatments within blocks. Prior to treatment application, we assessed plant trait uniformity (stem length, number of leaves, leaf length, chlorophyll, and FA) among the groups. We measured the leaf parameters from two expanded leaves per plant (n = 320 leaves), representing half of the number of leaves of each individual. From now on, we refer to these leaves as “1st week leaves”. All traits were similar among groups (GLM: *p* > 0.09) (Table S1).

To implement belowground competition, we planted two *T. diversifolia* individuals approximately 2 cm apart in the same pot, whereas control plants were grown individually (Figure S2a). We manipulated soil enrichment by applying approximately 12 g of a slow-release fertilizer (15N:9P:12K, Osmocote ForthCote Plus) to the substrate surface of treatment plants, while control plants remained unfertilized (Figure S2b,c). Finally, we simulated herbivory over two weeks by manually removing leaf tissue with scissors. Although biocontrol agents can completely defoliate *T. diversifolia* in the field [105], we applied moderate damage, following protocols used for other Asteraceae (e.g., *Solidago canadensis* [106]), to allow trait assessment of damaged leaves over the two-week period. In the first week, we removed approximately 50% of the leaf area from four leaves per plant; in the second week, we removed an additional 50% of leaf area from half of the newly expanded leaves (1–3 leaves per plant, Figure S2c). During the first week, plants had one-lobed leaves, and by the second week leaves ranged from one to three lobes.No further damage was applied after the second week to allow new leaves to develop until the end of the experiment. Control plants remained undamaged (Figure S2d). Removed leaf tissue was stored at −1 °C and later included in aboveground biomass measurements.

In the second week of the experiment, we marked the apical bud region of each plant stem with a permanent marker to allow trait measurements of newly developed leaves without confounding them with leaves sampled earlier. At the end of the experiment (four weeks; October–November 2016), we sampled four apical three-lobed leaves (50% of leaves per plant) for trait measurement, hereafter referred to as “4th week leaves”. By this time, some leaves marked during the first week (1st week leaves) had senesced and could not be resampled; however, the number of 1st week leaves sampled was similar among groups (*p* > 0.4). In addition, many plants in the herbivory and competition treatments had ceased stem growth and showed rapid leaf desiccation. Consequently, some plants had only two leaves in the 4th week, but the number of leaves sampled did not differ among groups (GLM: *p* > 0.2). These biological constraints naturally limited further accurate trait measurement (e.g., chlorophyll content); thus, we terminated the experiment at this time.

### 4.4. Trait Sampling

To test the effects in both experiments, we evaluated leaf and vegetative traits related to architecture, resource use, and stress in *T. diversifolia*. Architectural and biomass traits included stem length (cm), number of leaves, plant biomass (mg), shoot biomass, root/shoot biomass ratio, and relative growth rate (RGR). Leaf traits included leaf chlorophyll content (SPAD units) and leaf mass per area (LMA, g mm^−2^). We also assessed leaf FA as a stress indicator [107,108]. These traits are highly plastic and reflect plant responses to environmental conditions [109,110,111]. Specifically, we used architecture and biomass traits as indicators of plant growth and performance, leaf chlorophyll as a proxy for photosynthetic capacity, and LMA as an indicator of anti-herbivory defense.

To quantify architectural traits, we measured stem length from the soil surface to the apical bud using a measuring tape and counted the number of expanded non-senescent leaves of each plant. We estimated RGR of plants as the difference in natural logarithms of stem length between 4th and 1st week divided by time interval in days $\left(\Delta RGR=\;\frac{lnln\;\left(length\;4th\;week\right)\;-\;lnln\;\left(length\;1st\;week\right)\;}{\Delta t}\right)$ [112]. At the end of the experiment, we assessed biomass traits by oven-drying the above- and belowground parts of the plants at 60 °C for 48 h and weighing them separately in a precision balance (0.0001 g, M214ai Bel [Bel Engineering^®^, Monza, Italy]). We calculated root/shoot biomass ratio as belowground divided by aboveground biomass.

We assessed leaf traits from 1st and 4th week leaves of each plant in both experiments. With this approach, we aimed to capture temporal variation in leaf traits, as leaves at different developmental stages may respond differently to environmental stressors and resource availability, while also increasing the variability and representativeness of the sampled traits [56]. We measured leaf chlorophyll content at the middle portion of each leaf using the SPAD 502 Plus chlorophyll meter (Konica Minolta^®^, Tokyo, Japan), which provides rapid, non-destructive estimates of chlorophyll based on light absorption [113]. We standardized measurements of both leaf groups to the third week of leaf development to account for chlorophyll ontogenetic changes [114]. To estimate leaf sclerophylly, we assessed leaf area (mm^2^) in a flat scanner (600 dpi, Laser Jet Pro [HP^®^, Palo Alto, CA, USA]) and measured the images in the ImageJ software [115]. We dried and weighed leaf mass (mg) using the same method previously described for plant biomass. We calculated LMA as the ratio of dry mass to leaf area [116]. In experiment 2, we did not measure leaf area of 1st week leaves because the herbivory caused extensive blade damage, preventing accurate area estimation. Therefore, we evaluated sclerophylly only in the 4th week leaves in this experiment.

We quantified leaf FA using 1st and 4th week leaves from both experiments. To do so, we flattened each leaf between transparent glass plates with a millimetric scale (Figure S1a,b) and positioned a digital camera (Nikon^®^ D3200, Tokyo, Japan) perpendicularly above them to capture the images. We performed all measurements before leaf detachment, during the fourth week of development for both leaf groups, when the leaves were fully expanded. From the images, we measured the distance (mm) from the midvein to the right side (Rs) and the left side (Ls) leaf margins at the middle of each leaf blade to further calculate FA (Figure S3; [117]). Additionally, we assessed leaf length (mm) to evaluate FA assumptions [118]. We performed all measurements with the ImageJ version 1.5 software [115]. To minimize measurement bias, we randomized the order of the images, and the same researcher measured each leaf twice with a minimum interval of seven days [119].

### 4.5. Statistical Analysis

We ran all analyses in R_4.3.0_ [120], using the glmmTMB_1.1.9_ package [121] to fit the linear models comparing treatment levels. We evaluated model assumptions with the DHARMa_0.4.7_ package [122]. We explored significant treatment interactions using pairwise comparisons using the emmeans package [123]. We performed all analyses separately for each experiment.

#### 4.5.1. Architecture and Biomass Accumulation Traits

In experiment 1, we evaluated the isolated and combined effects of herbivory (undamaged vs. damaged) and shading condition (shaded vs. no shade). We used GLMs to test the effect of herbivory and shading conditions, and their interaction (independent variables), on seedling architectural traits (stem length, number of leaves, and relative growth rate) and on shoot and root/shoot biomass (dependent variables). We adopted the Gaussian distribution in all models and lognormal distribution for the number of leaves. In experiment 2, we tested the individual and combined effects of herbivory (undamaged vs. damaged), soil fertilization (unfertilized vs. fertilized), and belowground competition (isolated vs. competing). We used GLMs in which architectural traits and shoot and root/shoot biomass were treated as dependent variables, whereas herbivory, fertilization, and competition levels and their interactions were treated as independent variables. We fitted all models with the Gaussian distribution, except for the number of leaves (lognormal distribution) and shoot biomass (*t*-distribution). We used Tukey post hoc tests to compare significant interactions among factors in both experiments.

#### 4.5.2. Leaf Traits

To investigate the effect of herbivory, shading, and their interaction on leaf traits (chlorophyll content and LMA) in experiment 1, we used generalized linear mixed models (GLMMs) followed by Tukey post hoc tests. Leaf traits were treated as dependent variables, herbivory and shading as fixed factors, leaf sampling week (1st vs. 4th) as an additional fixed factor, and plant identity as a random effect [124]. We analyzed leaf chlorophyll using a Gaussian distribution and LMA using *t*-distribution (identity link). In experiment 2, we tested the effect of herbivory, fertilization, competition, and their interactions (independent variables) on leaf chlorophyll (dependent variable) using GLMMs with Gaussian distribution, considering leaf sampling week as a random effect. Because LMA could not be measured for 1st week leaves due to herbivory, this trait was analyzed only for 4th week leaves. Thus, we used GLM with a t-distribution, including LMA as dependent variable and herbivory, competition, fertilization, and their interactions as independent variables. Significant interactions among factors were assessed with Tukey post hoc test.

#### 4.5.3. Fluctuating Asymmetry

We performed the FA analyses separately for leaves sampled in the 1st and 4th weeks in both experiments. Following [125], we verified the assumptions for developmental instability studies, including leaf width measurement reliability, absence of antisymmetry or directional asymmetry, outlier presence, and allometric effects on FA [40,118]. These assumptions ensure that FA results reflect true environmental stressors rather than genetic or measurement bias [126].

To test measurement precision and directional asymmetry presence in leaf width, we used linear mixed models (LMMs), with leaf width as the dependent variable, leaf size as fixed effect, and leaf identity nested within replication as random effect. Low measurement error on data is confirmed when the variance component of leaf sides and leaf identity (Mv) is much higher than the measurement error (ME) [125]. Directional asymmetry is detected when one side of the leaf is significantly higher than the other [127]. We evaluated antisymmetry using Kolmogorov–Smirnov normality tests followed by histograms on leaf width side differences (Rs–Ls). A deviation from a Gaussian distribution indicates antisymmetry [128]. We checked for outliers in leaf width FA data using boxplots and Grubbs’ tests. Outlier presence may distort FA variation and lead to misleading interpretations [40]. Finally, to evaluate the allometric effect of size on FA, we used linear models (LMs) relating leaf length (independent variable) and leaf width FA (dependent variable). Allometric effects may bias FA, potentially leading to misleading interpretations [129].

We present the results of FA assumption tests for all groups in both experiments. Measurement errors were below 3% of the variation between leaf sides (ME < Mv), indicating high precision in leaf width measurements. Furthermore, LMMs showed no evidence of directional asymmetry (*p* > 0.1 in all cases), and antisymmetry was excluded based on normality tests (*p* > 0.3) and mesokurtic histograms. Regarding outlier evaluation, boxplots and Grubbs’ tests revealed no significant outliers (*p* > 0.07). However, an allometric effect of leaf length on FA width was detected in both experiments (R^2^ > 0.8, *p* < 0.001). To correct for this size dependence, we calculated the mean FA for each plant in each experiment using the “Index 3”: $AF=\;\frac{\sum \left[\frac{\left|Rs-Ls\right|}{\;(Rs+Ls)/2}\right]}{n}$ [108,118].

To evaluate the effects of herbivory and shading (experiment 1), and herbivory, competition, and fertilization (experiment 2) (independent variables), and their interactions on leaf FA (dependent variable), we used GLMMs with Gaussian distributions. Treatments and leaf sampling week (1st vs. 4th) were included as fixed effects, and plant identity as random effect [124]. Significant interactions were compared with Tukey post hoc tests.

## 5. Conclusions

Our greenhouse experiments suggest that isolated and combined biotic and abiotic pressures drive distinct performance and plastic responses in *T. diversifolia*, highlighting the role of phenotypic plasticity in the persistence of invasive plants across controlled heterogeneous conditions. The responses of simulated combined factors varied from performance declines to tolerance, indicating that invasion success depends on context-dependent trait adjustments rather than on single stressors. Our findings emphasize that management strategies for plant invasion, especially those based on herbivores, should consider local environmental conditions, as resource availability and competition can strongly modulate their effectiveness. For instance, herbivore biocontrol may be more effective on invasive plants under intense intraspecific competition than on those under low competition or growing in nutrient-rich soils. Regarding FA, our study does not consistently associate this bioindicator with plant negative performance under isolated and associated simulated stressors. However, it is also important to note that FA responses may vary by species, trait, or scale, which could explain some of the inconsistencies observed in our experiments. Thus, FA should be applied with caution as a biomarker of stress in invasive plants, especially in studies involving multiple interacting environmental factors. Overall, our experimental study advances knowledge in invasion ecology by providing evidence of how interactions among multiple biotic and abiotic factors shape the performance and plasticity of invasive plants under controlled heterogeneous conditions.

## Figures and Tables

**Table 1:** GLM results for the effects of herbivory, shade condition, and their interaction on vegetative traits of *Tithonia diversifolia* (experiment 1). Significant values (*p* < 0.05) are in bold.

Trait	Factor	χ^2^	df	*p*-Value
Stem length (cm)	Herbivory	0.02	1	0.853
	Shade condition	14.84	1	**<0.001**
	Herb × Shade	0.04	1	0.849
Number of leaves	Herbivory	1.64	1	0.2
	Shade condition	23.87	1	**<0.001**
	Herb × Shade	10.95	1	**<0.001**
Root/shoot biomass	Herbivory	0.01	1	0.91
	Shade condition	55.44	1	**<0.001**
	Herb × Shade	0.66	1	0.42
Relative growth rate	Herbivory	0.11	1	0.74
	Shade condition	13.51	1	**<0.001**
	Herb × Shade	0.04	1	0.84
Shoot biomass	Herbivory	0.14	1	0.709
	Shade condition	114.03	1	**<0.001**
	Herb × Shade	0.28	1	0.593

**Table 2:** Effects of herbivory, shade condition, and study weeks on leaf traits of *Tithonia diversifolia*. Significant GLMM results (*p* < 0.05) are shown in bold. Legends: LMA = leaf mass per area, FA = fluctuating asymmetry.

Trait	Factor	χ^2^	df	*p*-Value
Chlorophyll (SPAD units)	Herbivory	0.03	1	0.852
	Shade condition	0.707	1	0.4
	Week	100.56	1	**<0.001**
	Herb × Shade	0.012	1	0.729
	Herb × Week	0.85	1	0.356
	Shade × Week	15.89	1	**<0.001**
	Herb × Shade × Week	5.04	1	**0.025**
LMA (g mm^−2^)	Herbivory	5.41	1	**0.02**
	Shade condition	287.09	1	**<0.001**
	Week	1.94	1	0.163
	Herb × Shade	2.03	1	0.155
	Herb × Week	3.23	1	0.072
	Shade × Week	64.28	1	**<0.001**
	Herb × Shade × Week	2.49	1	0.115
FA (mm)	Herbivory	0.01	1	0.953
	Shade condition	3.86	1	**0.049**
	Week	2.02	1	0.155
	Herb × Shade	0.25	1	0.614
	Herb × Week	0.03	1	0.857
	Shade × Week	5.99	1	**0.014**
	Herb × Shade × Week	3.68	1	0.055

**Table 3:** Results of GLM showing the effects of herbivory, belowground competition, and fertilization on vegetative traits of *Tithonia diversifolia*. Significant results (*p* < 0.005) are highlighted in bold.

Trait	Factor	χ^2^	df	*p*-Value
Stem length (cm)	Herbivory	6.18	1	**0.013**
	Competition	1.51	1	0.22
	Fertilization	85.01	1	**<0.001**
	Herb × Comp	0.77	1	0.39
	Herb × Fert	0.92	1	0.338
	Comp × Fert	0.41	1	0.52
	Herb × Comp × Fert	1.18	1	0.278
Number of leaves	Herbivory	16.95	1	**<0.001**
	Competition	30.81	1	**<0.001**
	Fertilization	119.42	1	**<0.001**
	Herb × Comp	0.01	1	0.954
	Herb × Fert	0.01	1	0.93
	Comp × Fert	0.04	1	0.851
	Herb × Comp × Fert	0.02	1	0.866
Root/shoot biomass	Herbivory	11.61	1	**<0.001**
	Competition	0.01	1	0.93
	Fertilization	131.98	1	**<0.001**
	Herb × Comp	0.01	1	0.968
	Herb × Fert	12.22	1	**<0.001**
	Comp × Fert	4.27	1	**0.004**
	Herb × Comp × Fert	2.43	1	0.119
Relative growth rate	Herbivory	4.36	1	**0.037**
	Competition	2.23	1	0.136
	Fertilization	63.26	1	**<0.001**
	Herb × Comp	1.7	1	0.192
	Herb × Fert	1.1	1	0.294
	Comp × Fert	0.02	1	0.877
	Herb × Comp × Fert	1.34	1	0.247
Shoot biomass	Herbivory	15.44	1	**<0.001**
	Competition	47.89	1	**<0.001**
	Fertilization	316.63	1	**<0.001**
	Herb × Comp	0.14	1	0.7
	Herb × Fert	9.58	1	**0.002**
	Comp × Fert	62.62	1	**<0.001**
	Herb × Comp × Fert	1.33	1	0.25
LMA (g mm^−2^)	Herbivory	1.17	1	0.28
	Competition	13.48	1	**<0.001**
	Fertilization	10.78	1	**0.001**
	Herb × Comp	2.02	1	0.156
	Herb × Fert	4.01	1	**0.045**
	Comp × Fert	1.55	1	0.213
	Herb × Comp × Fert	0.92	1	0.337

**Table 4:** GLMM results for leaf chlorophyll content and fluctuating asymmetry (FA) of *Tithonia diversifolia* in response to simulated herbivory, belowground competition, and edaphic fertilization. Significant effects (*p* < 0.05) are shown in bold. Legend: FA = fluctuating asymmetry.

Trait	Factor	χ^2^	df	*p*-Value
Chlorophyll (SPAD units)	Herbivory	2.02	1	0.156
	Competition	3.89	1	**0.048**
	Fertilization	99.1	1	**<0.001**
	Week	115.14	1	**<0.001**
	Herb × Comp	0.01	1	0.967
	Herb × Fert	2.79	1	0.095
	Comp × Fert	1.96	1	0.162
	Herb × Week	0.29	1	0.589
	Comp × Week	1.94	1	0.164
	Fert × Week	191.42	1	**<0.001**
	Herb × Comp × Fert	0.03	1	0.843
	Herb × Comp × Week	7.14	1	**0.008**
	Herb × Fert × Week	2.33	1	0.127
	Comp × Fert × Week	0.11	1	0.745
	Herb × Comp × Fert × Week	1.24	1	0.265
FA (mm)	Herbivory	0.57	1	0.45
	Competition	3.06	1	**0.08**
	Fertilization	8.2	1	**0.004**
	Week	88.83	1	**<0.001**
	Herb × Comp	0.18	1	0.675
	Herb × Fert	5.67	1	**0.017**
	Comp × Fert	2.56	1	0.11
	Herb × Week	0.3	1	0.585
	Comp × Week	0.1	1	0.752
	Fert × Week	4.22	1	**0.04**
	Herb × Comp × Fert	4.74	1	0.09
	Herb × Comp × Week	0.03	1	0.867
	Herb × Fert × Week	11.54	1	**<0.001**
	Comp × Fert × Week	1.98	1	0.159
	Herb × Comp × Fert × Week	0.03	1	0.843

## Data Availability

The raw data of the study will be made available by the authors on request.

## Supplementary Materials

The following supporting information can be downloaded at https://www.mdpi.com/article/10.3390/plants15030349/s1, Figure S1: General view of experiment 1 conducted on *Tithonia diversifolia* under greenhouse conditions. Herbivory treatments: (A) Control (no damage) and (B) simulated herbivory (two circular damages). Details of the pair of glass plates used to capture images of the leaves. Shading (shade condition) treatments: (C) Control (no shade) and (D) competition (shade). E) View of the plants inside the shade cloth; Figure S2: *Tithonia diversifolia* from experiment 2 under greenhouse condition. (A) Belowground competition treatments: Control (isolated, lower panel) and competing individuals (upper panel). Fertilization treatments: (B) Control (no fertilization) and (C) fertilized soil (NPK enrichment); white arrows indicate the NPK fertilizer capsules. Herbivory treatments: (D) Control (no damage) and (E) simulated herbivory (50% of leaf blade removed); yellow arrows indicate the damaged areas performed during the second week of the study; Figure S3: Leaf of *Tithonia diversifolia* showing the procedure used to assess fluctuating asymmetry at the central region of the leaf blade in experiments 1 and 2. Legend: Ls—Left side, Rs—Right side; Table S1: GLM results showing the similarity for vegetative, leaf traits, and fluctuating asymmetry (FA) of *Tithonia diversifolia* among treatment groups of experiment 1 and experiment 2. The data of each trait is presented as mean ± standard deviation ($\bar{X}$ ± SD). Legend: ns = non-significant.
