# Supplementary material for: Under Pressure: Shading, High Herbivory, and Low Levels of Fertilization Drive the Vegetative Response of a Highly Invasive Species
Source: Plants (Basel). 2026 Jan 23;15(3):349. doi: 10.3390/plants15030349 (PMC12899931; doi:10.3390/plants15030349)
Supplement: Supplementary file 1 [file plants-15-00349-s001.zip › plants-4074924-supplementary.pdf]

Tables

Table S1. GLM results showing the similarity for vegetative, leaf traits and fluctuating asymmetry (FA) of *Tithonia diversifolia* among treatment groups of experiment 1 and experiment 2. The data of each trait is presented as mean ± standard deviation ($\bar{X}$ ± SD). Legend: ns = non-significant.

Experiment	Trait	$\bar{X}$ ± SD	Factor	F-value
1	Stem length (cm)	25.14 ± 6.36	Herbivory	0.26^{ns}
			Shade condition	0.36 ^{ns}
			Herb x Shade	0.17 ^{ns}
	Number of leaves	7.09 ± 1.43	Herbivory	0.03 ^{ns}
			Shade condition	0.03 ^{ns}
			Herb x Shade	0.03 ^{ns}
	Leaf length (mm)	83.32 ± 20.73	Herbivory	0.27 ^{ns}
			Shade condition	0.21 ^{ns}
			Herb x Shade	0.59 ^{ns}
	Chlorophyll content (SPAD Units)	31.12 ± 6.25	Herbivory	0.22 ^{ns}
			Shade condition	0.56 ^{ns}
			Herb x Shade	0.26^{ns}
	Leaf FA (mm)	0.13 ± 0.06	Herbivory	0.01 ^{ns}
			Shade condition	0.03 ^{ns}
			Herb x Shade	2.29 ^{ns}
2	Stem length (cm)	4.17 ± 0.68	Herbivory	1.05 ^{ns}
			Competition	0.96 ^{ns}
			Fertilization	0.17 ^{ns}
			Herb x Comp	1.6 ^{ns}
			Herb x Fert	0.77 ^{ns}
			Comp x Fert	0.15 ^{ns}
			Herb x Comp x Fert	0.22 ^{ns}
	Number of leaves	4	-	-
	Leaf length (mm)	22.43 ± 4.97	Herbivory	1.1 ^{ns}
			Competition	0.91 ^{ns}
			Fertilization	0.03 ^{ns}
			Herb x Comp	2.57 ^{ns}
			Herb x Fert	0.6 ^{ns}
			Comp x Fert	0.02 ^{ns}
			Herb x Comp x Fert	0.05 ^{ns}
	Chlorophyll content (SPAD Units)	28.43 ± 3.45	Herbivory	0.55^{ns}
			Competition	0.36 ^{ns}
			Fertilization	2.78 ^{ns}
			Herb x Comp	3.3 ^{ns}
			Herb x Fert	0.08 ^{ns}
			Comp x Fert	0.76 ^{ns}
			Herb x Comp x Fert	0.35 ^{ns}
	Leaf FA (mm)	0.07 ± 0.03	Herbivory	0.25 ^{ns}
			Competition	0.21 ^{ns}
			Fertilization	0.48 ^{ns}
			Herb x Comp	0.61 ^{ns}
			Herb x Fert	0.371 ^{ns}
			Comp x Fert	0.868 ^{ns}

Herb x Comp x Fert	0.699 ^{ns}

Figures

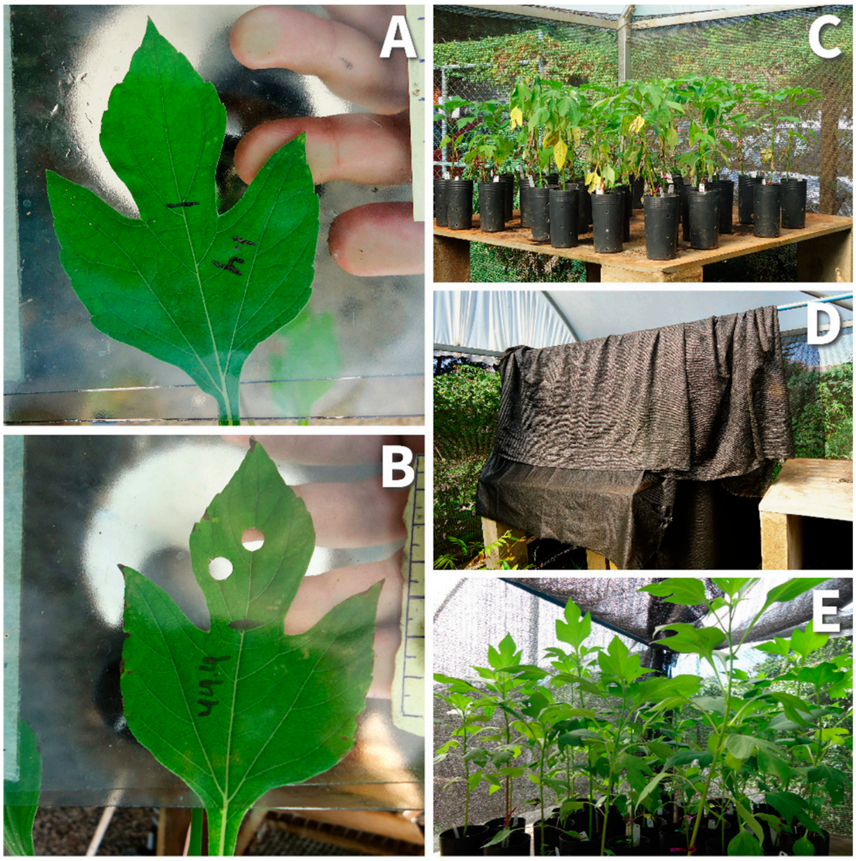

Figure S1. General view of experiment 1 conducted on *Tithonia diversifolia* under greenhouse conditions. Herbivory treatments: A) control (no damage) and B) simulated herbivory (two circular damages). Details of the pair of glass plates used to capture images of the leaves. Aboveground competition (Shade condition) treatments: C) control (no shade) and D) competition (shade). E) View of the plants inside the shade cloth.

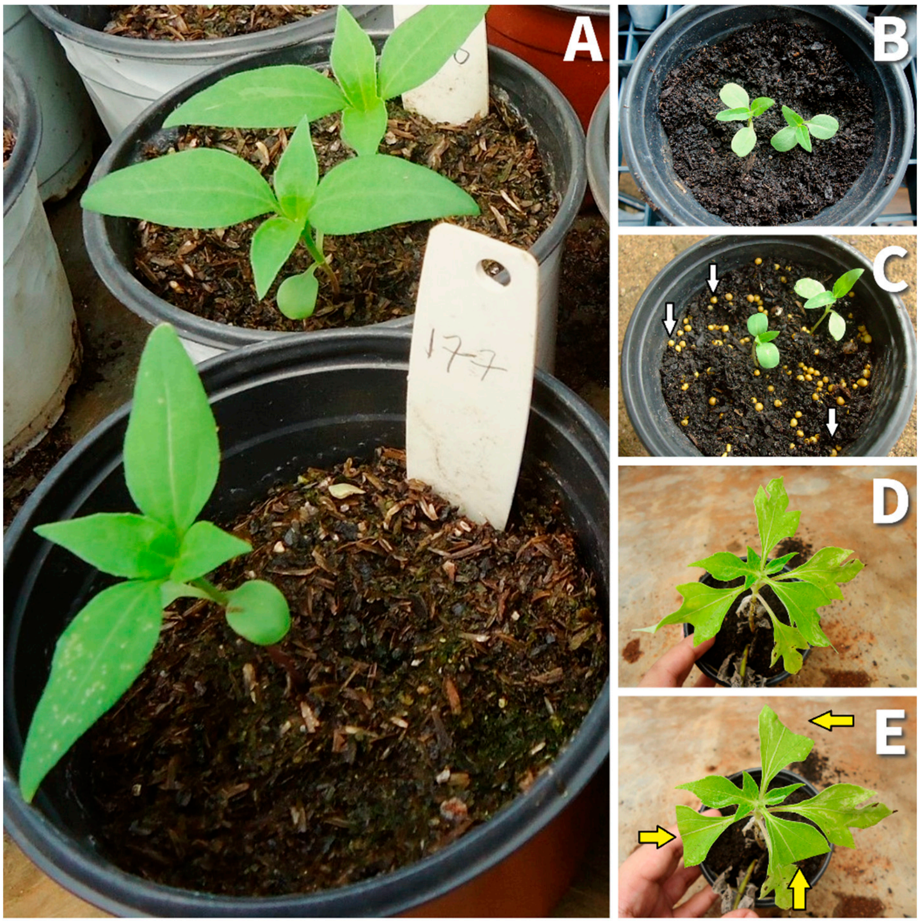

Figure S2. *Tithonia diversifolia* from experiment 2 under greenhouse condition. A) Belowground competition treatments: control (isolated, lower panel) and competing individuals (upper panel). Fertilization treatments: B) control (no fertilization) and C) fertilized soil (NPK enrichment); white arrows indicate the NPK fertilizer capsules. Herbivory treatments: D) control (no damage) and simulated E) herbivory (50% of leaf blade removed); yellow arrows indicate the damaged areas performed during the second week of the study.

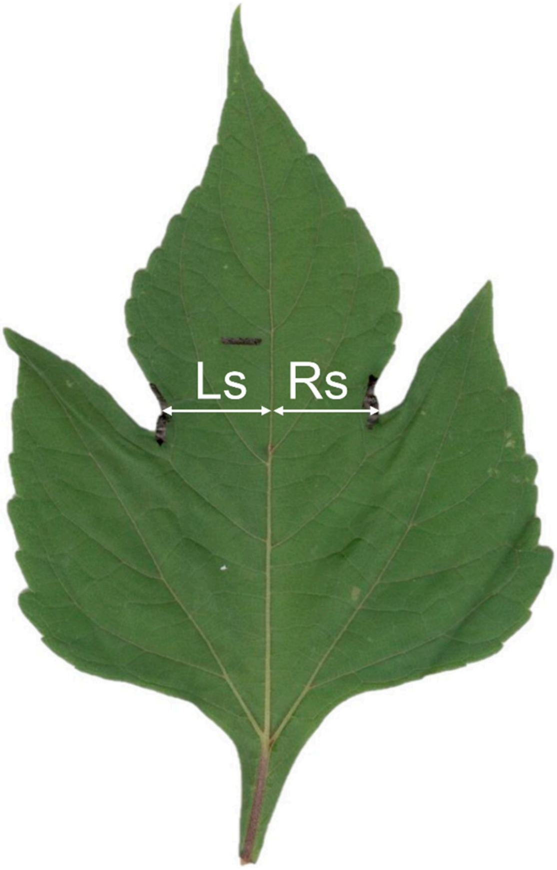

Figure S3. Leaf of *Tithonia diversifolia* showing the procedure used to assess fluctuating asymmetry at the central region of the leaf blade in experiments 1 and 2. Legend: Ls – Left side, Rs – Right side.
